# Comorbid adult adhd and bipolar affective disorder – assessment challenges

**DOI:** 10.1192/j.eurpsy.2021.515

**Published:** 2021-08-13

**Authors:** I. Pereira, V. Nogueira, M. Marguilho, J. Teixeira

**Affiliations:** 1 Clínica 4 - Unidade De Alcoologia E Novas Dependências, Centro Hospitalar Psiquiátrico de Lisboa, Lisboa, Portugal; 2 Clínica 5, Centro Hospitalar Psiquiátrico de Lisboa, Lisboa, Portugal

**Keywords:** attention deficit and hyperactivity disorder, comorbidity in adult adhd, bipolar affective disorder

## Abstract

**Introduction:**

Attention deficit and hyperactivity disorder (ADHD) and bipolar disorder (BD) are two of the most prevalent psychiatric disorders presenting in children and adults, respectively. Reported co-occurrence of ADHD and BD in adulthood is higher than would be expected by chance, with great impact on prognosis and treatment. Since features of both entities can overlap, careful assessment of these patients is crucial.

**Objectives:**

To understand the relation between BD and ADHD, and how co-occurrence impacts clinical evaluation.

**Methods:**

Bibliographic research was made through the PubMed/NCBI database. No time limit was specified on the search. Pertinent manuscripts were individually reviewed for additional relevant citations.

**Results:**

ADHD influences the course and manifestations of BD, regardless of its presence later in adulthood. There is a 3-fold increase of ADHD co-occurrence in individuals with BD when compared to normal population, and ADHD seems to co-occur in about 20% of BD patients (even after correction for overlapping symptoms). Features which may suggest simultaneous diagnosis are: earlier occurrence of BD-related symptoms (especially manic or hypomanic states), more severe course of the mood disorder, less adherence to treatment and higher functioning impact. This makes for a worse prognosis, with increased suicidal risk in these patients.

**Conclusions:**

The co-occurrence of BD and ADHD may represent a distinct clinical phenotype, with recent findings highlighting the presence of common neurobiological mechanisms. Accordingly, patients with BD should be screened for ADHD and viceversa. There is no consensus for treatment of ADHD-BD patients, with further studies being necessary to better define and define possible therapeutic approaches.

